# Supplementary material for: MetaRibo-Seq measures translation in microbiomes
Source: Nat Commun. 2020 Jun 29;11:3268. doi: 10.1038/s41467-020-17081-z (PMC7324362; doi:10.1038/s41467-020-17081-z)
Supplement: Supplementary file 10 — Supplementary Data 7 [file 41467_2020_17081_MOESM10_ESM.zip › File2/Confidence_High_Taxonomy/108895_out.krona.html]

Javascript must be enabled to view this page.

members
magnitude
magnitudeUnassigned
count
unassigned
taxon
rank

108895\_out

18

2
18
superkingdom

18
phylum
1224

18
class
1236

order
18
91347

18
543
2

SRS046502\_contig\_number\_contig-100\_23615.66940SRS143565\_contig\_number\_contig-100\_12802.44580
family

3
561
16
genus

SRS012902\_contig\_number\_contig-100\_4177.4177SRS063127\_contig\_number\_contig-100\_1736.1737SRS140492\_contig\_number\_contig-100\_3412.181945

562
species
13

SRS012273\_contig\_number\_contig-100\_1117.270378SRS017191\_contig\_number\_contig-100\_365.124682SRS019129\_contig\_number\_contig-100\_1039.100527SRS045244\_contig\_number\_contig-100\_18642.18643SRS046502\_contig\_number\_contig-100\_8708.52033SRS064276\_contig\_number\_35325SRS077127\_contig\_number\_contig-100\_12638.12639SRS077454\_contig\_number\_contig-100\_5883.97433SRS144362\_contig\_number\_45851SRS146888\_contig\_number\_15707SRS146888\_contig\_number\_contig-100\_1090.100211SRS147022\_contig\_number\_14770SRS148091\_contig\_number\_contig-100\_1474.1475
